# 2-(2*H*-Tetra­zol-5-yl)pyridinium nitrate

**DOI:** 10.1107/S1600536809018078

**Published:** 2009-05-20

**Authors:** Li-Jing Cui, Miao-Jia Yu

**Affiliations:** a Ordered Matter Science Research Center, College of Chemistry and Chemical Engineering, Southeast University, Nanjing 210096, People’s Republic of China

## Abstract

In the cation of the title compound, C_6_H_6_N_5_^+^·NO_3_^−^, the dihedral angle between the pyridinium and tetra­zole rings is 8.2 (2)°. The constituent ions of the compound are linked *via* N—H⋯O hydrogen bonds, forming helical chains running along the *b* axis. C—H⋯N and C—H⋯O hydrogen bonds are also observed.

## Related literature

For the use of tetra­zole derivatives in coordination chemistry, see: Xiong *et al.* (2002 ▶); Fu *et al.* (2008 ▶); Wang *et al.* (2005 ▶). For the crystal structures of related compounds, see: Dai & Fu (2008 ▶); Wen (2008 ▶).
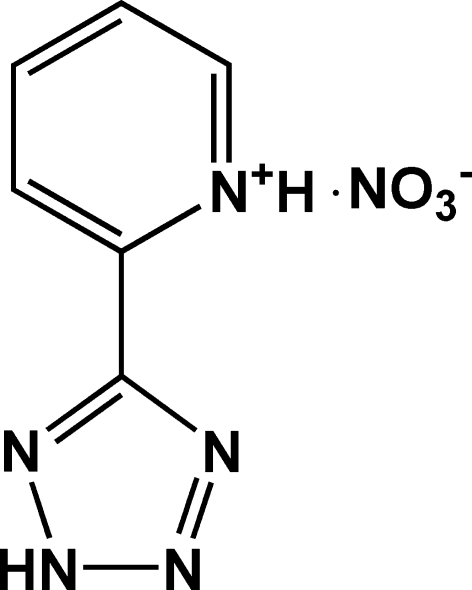


## Experimental

### 

#### Crystal data

C_6_H_6_N_5_^+^·NO_3_^−^
*M* *_r_* = 210.17
Monoclinic, 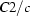

*a* = 20.400 (4) Å
*b* = 4.8981 (10) Å
*c* = 19.135 (4) Å
β = 114.77 (3)°
*V* = 1736.1 (7) Å^3^
*Z* = 8
Mo *K*α radiation
μ = 0.13 mm^−1^
*T* = 298 K
0.20 × 0.15 × 0.15 mm

#### Data collection

Rigaku Mercury2 diffractometer
Absorption correction: multi-scan (*CrystalClear*, Rigaku, 2005 ▶) *T*_min_ = 0.976, *T*_max_ = 0.980
8427 measured reflections
1999 independent reflections
1033 reflections with *I* > 2σ(*I*)
*R*_int_ = 0.123

#### Refinement

*R*[*F* ^2^ > 2σ(*F* ^2^)] = 0.081
*wR*(*F* ^2^) = 0.209
*S* = 1.04
1999 reflections
136 parameters
H-atom parameters constrained
Δρ_max_ = 0.34 e Å^−3^
Δρ_min_ = −0.36 e Å^−3^

### 

Data collection: *CrystalClear* (Rigaku, 2005 ▶); cell refinement: *CrystalClear*; data reduction: *CrystalClear*; program(s) used to solve structure: *SHELXS97* (Sheldrick, 2008 ▶); program(s) used to refine structure: *SHELXL97* (Sheldrick, 2008 ▶); molecular graphics: *SHELXTL* (Sheldrick, 2008 ▶); software used to prepare material for publication: *SHELXTL*.

## Supplementary Material

Crystal structure: contains datablocks I, global. DOI: 10.1107/S1600536809018078/ci2780sup1.cif

Structure factors: contains datablocks I. DOI: 10.1107/S1600536809018078/ci2780Isup2.hkl

Additional supplementary materials:  crystallographic information; 3D view; checkCIF report

## Figures and Tables

**Table 1:** Hydrogen-bond geometry (Å, °)

*D*—H⋯*A*	*D*—H	H⋯*A*	*D*⋯*A*	*D*—H⋯*A*
N1—H1⋯O3^i^	0.86	1.92	2.772 (4)	172
N4—H4*A*⋯O3	0.86	1.87	2.717 (4)	170
C2—H2⋯N2^ii^	0.93	2.58	3.507 (5)	173
C3—H3⋯O1^iii^	0.93	2.52	3.435 (5)	171
C5—H5⋯O2^i^	0.93	2.54	3.218 (5)	130
C5—H5⋯O2^iv^	0.93	2.36	3.223 (5)	155

Symmetry codes: (i) 
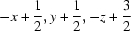
; (ii) 
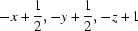
; (iii) 
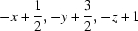
; (iv) 
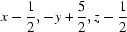
.

**Fig. 1.**
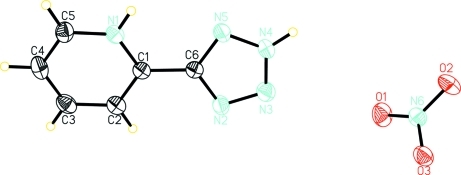
A view of the title compound with the atomic numbering scheme. Displacement ellipsoids are drawn at the 30% probability level.

**Fig. 2.**
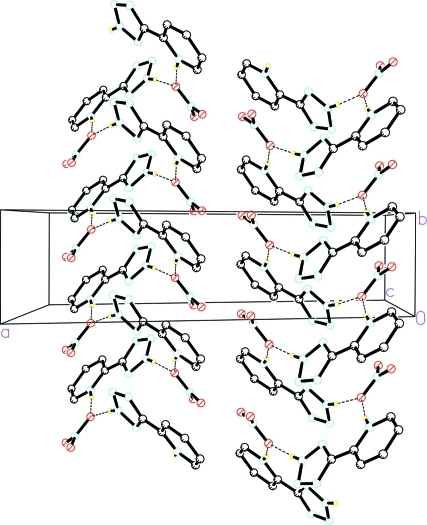
Partial packing view of the title compound showing the formation of chains along the b axis. H atoms not involved in hydrogen bonding (dashed lines) have been omitted for clarity.

## supplementary crystallographic information

### Comment

In the past few years, more and more people have focused on the chemistry of tetrazole derivatives because of their multiple coordination modes as ligands to metal ions and for the construction of novel metal-organic frameworks (Fu *et al.*, 2008; Wang, *et al.* 2005; Xiong, *et al.* 2002; Wen 2008). We report here the crystal structure of the title compound, 2-(2*H*-tetrazol-5-yl)pyridinium nitrate.

In the title compound (Fig.1), the N atom (N1) of the pyridine ring is protonated. The pyridine and tetrazole rings are nearly coplanar and are twisted from each other by a dihedral angle of 8.2 (2)°. The geometric parameters of the tetrazole ring are comparable to those observed in related structures (Wang *et al.* 2005; Dai & Fu 2008).

The crystal packing is stabilized by N—H···O hydrogen bonds which link the molecules into a helical chain running along the *b* axis (Table 1 and Fig.2). In addition, C—H···N and C—H···O hydrogen bonds are observed.

### Experimental

Picolinonitrile (30 mmol), NaN_3_ (45 mmol), NH_4_Cl (33 mmol) and DMF (50 ml) were added in a flask under nitrogen atmosphere. The mixture stirred at 110°C for 20 h. The resulting solution was then poured into ice-water (100 ml), and a white solid was obtained after adding HCl (6 *M*) at pH = 6. The precipitate was filtered and washed with distilled water. Colourless block-shaped crystals suitable for X-ray analysis were obtained from the crude product by slow evaporation of an ethanol-HNO_3_ (50:1 *v*/*v*) solution.

### Refinement

All H atoms attached to C and N atoms were fixed geometrically and treated as riding, with C–H = 0.93 Å, N–H = 0.86 Å and *U*_iso_(H) =1.2U_eq_(C or N).

### Crystal data

**Table:**

C_6_H_6_N_5_^+^·NO_3_^−^	*F*(000) = 864
*M**_r_* = 210.17	*D*_x_ = 1.608 Mg m^−^^3^
Monoclinic, *C*2/*c*	Mo *K*α radiation, λ = 0.71073 Å
Hall symbol: -C 2yc	Cell parameters from 1999 reflections
*a* = 20.400 (4) Å	θ = 3.8–27.5°
*b* = 4.8981 (10) Å	µ = 0.13 mm^−^^1^
*c* = 19.135 (4) Å	*T* = 298 K
β = 114.77 (3)°	Block, colourless
*V* = 1736.1 (7) Å^3^	0.20 × 0.15 × 0.15 mm
*Z* = 8	

### Data collection

**Table:**

Rigaku Mercury2 diffractometer	1999 independent reflections
Radiation source: fine-focus sealed tube	1033 reflections with *I* > 2σ(*I*)
graphite	*R*_int_ = 0.123
Detector resolution: 13.6612 pixels mm^-1^	θ_max_ = 27.5°, θ_min_ = 3.8°
ω scans	*h* = −26→26
Absorption correction: multi-scan (*CrystalClear*, Rigaku, 2005)	*k* = −6→6
*T*_min_ = 0.976, *T*_max_ = 0.980	*l* = −24→24
8427 measured reflections	

### Refinement

**Table:**

Refinement on *F*^2^	Primary atom site location: structure-invariant direct methods
Least-squares matrix: full	Secondary atom site location: difference Fourier map
*R*[*F*^2^ > 2σ(*F*^2^)] = 0.081	Hydrogen site location: inferred from neighbouring sites
*wR*(*F*^2^) = 0.209	H-atom parameters constrained
*S* = 1.04	*w* = 1/[σ^2^(*F*_o_^2^) + (0.0838*P*)^2^] where *P* = (*F*_o_^2^ + 2*F*_c_^2^)/3
1999 reflections	(Δ/σ)_max_ = 0.001
136 parameters	Δρ_max_ = 0.34 e Å^−^^3^
0 restraints	Δρ_min_ = −0.36 e Å^−^^3^

### Special details

**Table:**

Geometry. All e.s.d.'s (except the e.s.d. in the dihedral angle between two l.s. planes) are estimated using the full covariance matrix. The cell e.s.d.'s are taken into account individually in the estimation of e.s.d.'s in distances, angles and torsion angles; correlations between e.s.d.'s in cell parameters are only used when they are defined by crystal symmetry. An approximate (isotropic) treatment of cell e.s.d.'s is used for estimating e.s.d.'s involving l.s. planes.
Refinement. Refinement of *F*^2^ against ALL reflections. The weighted *R*-factor *wR* and goodness of fit *S* are based on *F*^2^, conventional *R*-factors *R* are based on *F*, with *F* set to zero for negative *F*^2^. The threshold expression of *F*^2^ > σ(*F*^2^) is used only for calculating *R*-factors(gt) *etc*. and is not relevant to the choice of reflections for refinement. *R*-factors based on *F*^2^ are statistically about twice as large as those based on *F*, and *R*- factors based on ALL data will be even larger.

### Fractional atomic coordinates and isotropic or equivalent isotropic displacement parameters (Å^2^)

**Table:**

	*x*	*y*	*z*	*U*_iso_*/*U*_eq_	
N1	0.11658 (15)	0.9420 (6)	0.51774 (16)	0.0383 (7)	
H1	0.1239	1.0055	0.5623	0.046*	
C6	0.21176 (18)	0.6182 (7)	0.58420 (19)	0.0362 (8)	
N2	0.24979 (17)	0.3908 (6)	0.58539 (17)	0.0485 (8)	
C1	0.15732 (18)	0.7297 (7)	0.51387 (19)	0.0364 (8)	
N5	0.23230 (16)	0.7307 (6)	0.65302 (17)	0.0447 (8)	
C3	0.0917 (2)	0.7442 (8)	0.3768 (2)	0.0506 (10)	
H3	0.0831	0.6771	0.3283	0.061*	
N4	0.28398 (16)	0.5624 (6)	0.69580 (17)	0.0463 (8)	
H4A	0.3082	0.5846	0.7445	0.056*	
N3	0.29538 (17)	0.3572 (7)	0.65769 (18)	0.0518 (9)	
C4	0.0518 (2)	0.9615 (9)	0.3841 (2)	0.0520 (11)	
H4	0.0161	1.0411	0.3408	0.062*	
C2	0.14442 (19)	0.6270 (8)	0.4418 (2)	0.0436 (9)	
H2	0.1711	0.4798	0.4371	0.052*	
C5	0.0657 (2)	1.0571 (8)	0.4558 (2)	0.0446 (9)	
H5	0.0395	1.2039	0.4615	0.053*	
N6	0.41263 (16)	0.8821 (6)	0.85439 (18)	0.0434 (8)	
O3	0.37035 (14)	0.6773 (5)	0.84530 (13)	0.0533 (8)	
O2	0.44457 (14)	0.9758 (6)	0.91958 (15)	0.0570 (8)	
O1	0.41958 (16)	0.9759 (6)	0.79835 (16)	0.0672 (9)	

### Atomic displacement parameters (Å^2^)

**Table:**

	*U*^11^	*U*^22^	*U*^33^	*U*^12^	*U*^13^	*U*^23^
N1	0.0387 (17)	0.0438 (18)	0.0341 (16)	−0.0043 (14)	0.0169 (14)	−0.0018 (14)
C6	0.0362 (19)	0.037 (2)	0.038 (2)	−0.0036 (17)	0.0179 (16)	−0.0003 (16)
N2	0.0487 (19)	0.054 (2)	0.0364 (18)	0.0079 (16)	0.0115 (15)	−0.0008 (15)
C1	0.0372 (19)	0.039 (2)	0.036 (2)	−0.0052 (16)	0.0181 (17)	−0.0005 (16)
N5	0.0421 (18)	0.0480 (19)	0.0379 (18)	0.0021 (15)	0.0108 (14)	−0.0026 (15)
C3	0.057 (3)	0.058 (3)	0.038 (2)	−0.015 (2)	0.020 (2)	−0.0046 (19)
N4	0.0434 (18)	0.0510 (19)	0.0352 (17)	−0.0010 (16)	0.0073 (14)	−0.0014 (16)
N3	0.047 (2)	0.055 (2)	0.049 (2)	0.0072 (17)	0.0154 (17)	−0.0032 (16)
C4	0.041 (2)	0.068 (3)	0.038 (2)	−0.009 (2)	0.0078 (18)	0.009 (2)
C2	0.042 (2)	0.049 (2)	0.043 (2)	−0.0064 (18)	0.0212 (18)	−0.0027 (18)
C5	0.040 (2)	0.046 (2)	0.045 (2)	−0.0021 (17)	0.0155 (19)	0.0074 (18)
N6	0.0382 (18)	0.049 (2)	0.0414 (19)	0.0006 (15)	0.0156 (15)	−0.0023 (16)
O3	0.0543 (17)	0.0578 (17)	0.0413 (16)	−0.0174 (14)	0.0136 (13)	−0.0034 (13)
O2	0.0527 (18)	0.070 (2)	0.0460 (17)	−0.0192 (14)	0.0180 (14)	−0.0189 (14)
O1	0.077 (2)	0.080 (2)	0.0499 (17)	−0.0107 (17)	0.0314 (16)	0.0090 (16)

### Geometric parameters (Å, °)

**Table:**

N1—C5	1.329 (4)	C3—H3	0.93
N1—C1	1.352 (4)	N4—N3	1.318 (4)
N1—H1	0.86	N4—H4A	0.86
C6—N5	1.323 (4)	C4—C5	1.363 (5)
C6—N2	1.352 (4)	C4—H4	0.93
C6—C1	1.446 (5)	C2—H2	0.93
N2—N3	1.314 (4)	C5—H5	0.93
C1—C2	1.386 (5)	N6—O1	1.228 (3)
N5—N4	1.318 (4)	N6—O2	1.229 (4)
C3—C4	1.382 (5)	N6—O3	1.286 (4)
C3—C2	1.383 (5)		
			
C5—N1—C1	123.0 (3)	N5—N4—H4A	122.8
C5—N1—H1	118.5	N3—N4—H4A	122.8
C1—N1—H1	118.5	N2—N3—N4	106.0 (3)
N5—C6—N2	112.7 (3)	C5—C4—C3	118.9 (4)
N5—C6—C1	124.7 (3)	C5—C4—H4	120.5
N2—C6—C1	122.6 (3)	C3—C4—H4	120.5
N3—N2—C6	105.6 (3)	C3—C2—C1	119.8 (4)
N1—C1—C2	117.9 (3)	C3—C2—H2	120.1
N1—C1—C6	119.3 (3)	C1—C2—H2	120.1
C2—C1—C6	122.8 (3)	N1—C5—C4	120.5 (4)
N4—N5—C6	101.3 (3)	N1—C5—H5	119.8
C4—C3—C2	119.8 (4)	C4—C5—H5	119.8
C4—C3—H3	120.1	O1—N6—O2	122.7 (3)
C2—C3—H3	120.1	O1—N6—O3	119.3 (3)
N5—N4—N3	114.4 (3)	O2—N6—O3	118.0 (3)

### Hydrogen-bond geometry (Å, °)

**Table:**

*D*—H···*A*	*D*—H	H···*A*	*D*···*A*	*D*—H···*A*
N1—H1···O3^i^	0.86	1.92	2.772 (4)	172
N4—H4A···O3	0.86	1.87	2.717 (4)	170
C2—H2···N2^ii^	0.93	2.58	3.507 (5)	173
C3—H3···O1^iii^	0.93	2.52	3.435 (5)	171
C5—H5···O2^i^	0.93	2.54	3.218 (5)	130
C5—H5···O2^iv^	0.93	2.36	3.223 (5)	155

Symmetry codes: (i) −*x*+1/2, *y*+1/2, −*z*+3/2; (ii) −*x*+1/2, −*y*+1/2, −*z*+1; (iii) −*x*+1/2, −*y*+3/2, −*z*+1; (iv) *x*−1/2, −*y*+5/2, *z*−1/2.
